# The oncogenic roles of JC polyomavirus in cancer

**DOI:** 10.3389/fonc.2022.976577

**Published:** 2022-09-23

**Authors:** Hua-chuan Zheng, Hang Xue, Cong-yu Zhang

**Affiliations:** ^1^ Department of Oncology and Central Laboratory, The Affiliated Hospital of Chengde Medical University, Chengde, China; ^2^ Cancer Center, The First Affiliated Hospital of Jinzhou Medical University, Jinzhou, China

**Keywords:** JC polyomavirus, cancer, oncogenesis, signal pathway, virus replication, virus infection

## Abstract

JC polyomavirus (JCPyV) belongs to the human polyomavirus family. Based on alternative splicing, the early region encodes the large and small T antigens, while the late region encodes the capsid structural proteins (VP1, VP2, and VP3) and the agnoprotein. The regulatory transcription factors for JCPyV include Sp1, TCF-4, DDX1, YB-1, LCP-1, Purα, GF-1, and NF-1. JCPyV enters tonsillar tissue through the intake of raw sewage, inhalation of air droplets, or parent-to-child transmission. It persists quiescently in lymphoid and renal tissues during latency. Both TGF-β1 and TNF-α stimulates JCPyV multiplication, while interferon-γ suppresses the process. The distinct distribution of caspid receptors (α-2, 6-linked sialic acid, non-sialylated glycosaminoglycans, and serotonin) determines the infection capabilities of JCPyV virions, and JCPyV entry is mediated by clathrin-mediated endocytosis. In permissive cells, JCPyV undergoes lytic proliferation and causes progressive multifocal leukoencephalopathy, while its DNA is inserted into genomic DNA and leads to carcinogenesis in non-permissive cells. T antigen targets p53, β-catenin, IRS, Rb, TGF-β1, PI3K/Akt and AMPK signal pathways in cancer cells. Intracranial injection of T antigen into animals results in neural tumors, and transgenic mice develop neural tumors, lens tumor, breast cancer, gastric, Vater’s, colorectal and pancreatic cancers, insulinoma, and hepatocellular carcinoma. Additionally, JCPyV DNA and its encoded products can be detected in the brain tissues of PML patients and brain, oral, esophageal, gastric, colorectal, breast, cervical, pancreatic, and hepatocellular cancer tissues. Therefore, JCPyV might represent an etiological risk factor for carcinogenesis and should be evaluated for early prevention, diagnosis, and treatment of cancers.

## Introduction

**Figure 1:**
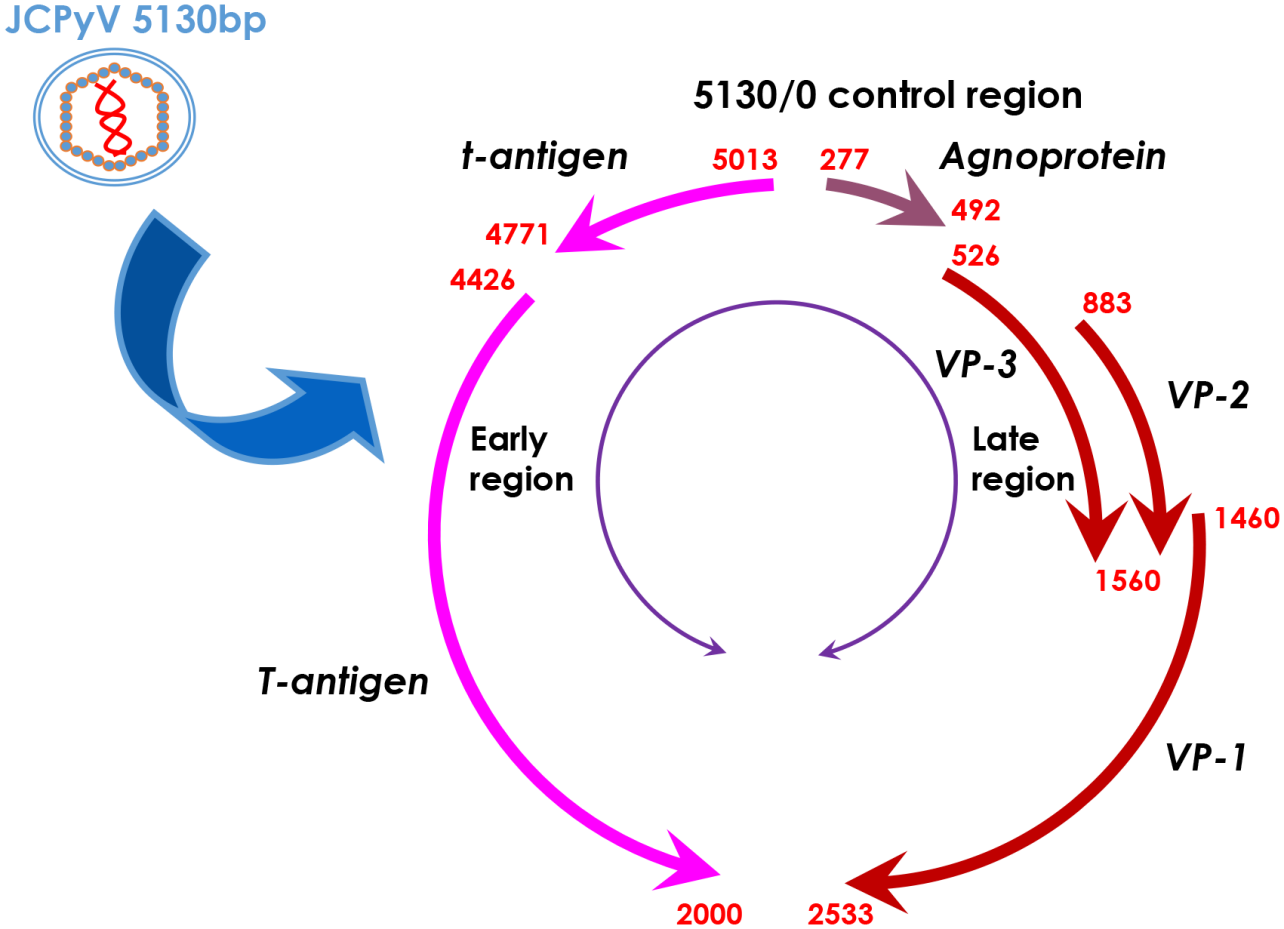
The genomic DNA structure of JCPyV. JCPyV has icosahedral capsids and small, circular, double-stranded genomic DNA of 5130 base pairs. It is composed of early and late coding regions, which are transcribed in opposite directions initiated by a transcriptional control region. The early region encodes both small and large T antigens by alternative splicing. The late region encodes the capsid structural proteins (VP1, VP2 and VP3) by alternative splicing and a small regulatory protein, agnoprotein.

**Figure 2:**
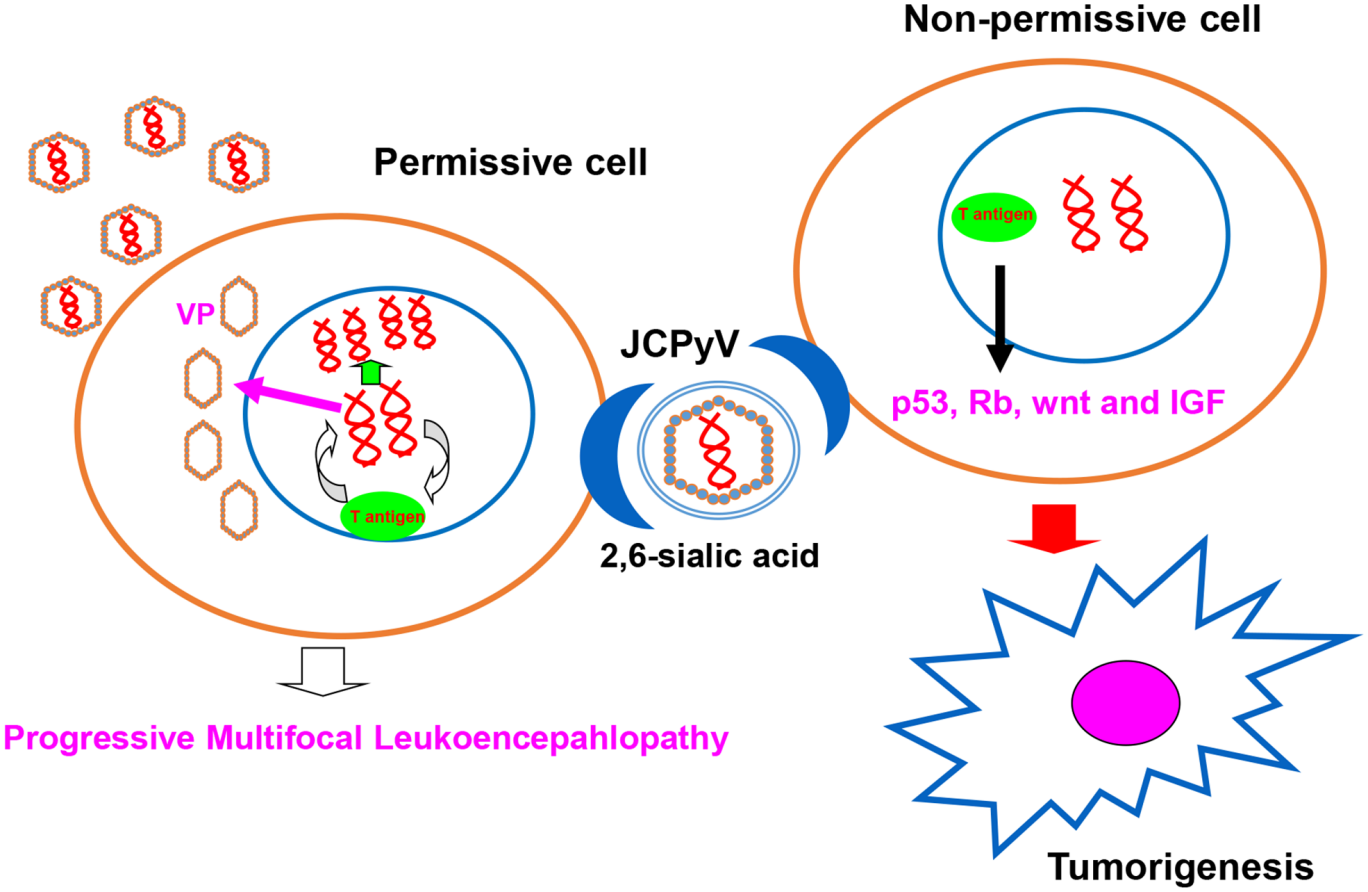
JCPyV infection outcome. JCPyV infection is initiated by its binding to JCPyV-sensitive cell surfaces. JCPyV capsids undergo endocytosis *via* capsid receptors (e.g., α 2, 6-linked sialic acid, non-sialylated glycosaminoglycans, and serotonin). In permissive cells, JCPyV may be activated for cell lysis and cause progressive multifocal leukoencephalopathy under immunosuppression (e.g., HIV infection, immunosuppressive drugs for organ transplantation, and cancer chemotherapy). In non-permissive cells, T antigen DNA from JCPyV can be inserted into genomic DNA, and T antigen can induce the malignant transformation of normal cells by targeting the p53, Rb, wnt, and IGF signal pathways.

JC polyomavirus (JCPyV) belongs to the human non-enveloped polyomavirus family in combination of SV40 and BK viruses. The genomic DNA homology between JCPyV and SV40 or BK viruses is 69% or 75%, respectively, showing their close evolutionary relationships (1). A serological study has indicated asymptomatic JCPyV infection in 46.1% of 1-month-old infants, 80.7% of 1- year-old infants, 85.9% of 2-year-old children, 85.5% of 3-year-old children, and about 90% of the adult population (2). As shown in **Figure 1**, JCPyV consists of a small, circular, double-stranded DNA genome of 5,130 base pairs and icosahedral capsids. The transcription of early and late coding regions occurs to produce small t and large T antigens by an interposed transcription control region. The late region encodes the capsid structural proteins (VP1, VP2, and VP3) by alternative splicing and a small regulatory protein (agnoprotein). T and t antigens are responsible for DNA replication, and the VP proteins for assemble with viral DNA to form virions (1). JCPyV may be activated for cell lysis under immunosuppressive conditions (e.g., HIV infection or the transplantation of bone marrow, liver, lung or kidney), and therefore is an established etiologic factor of demyelinating progressive multifocal leukoencephalopathy (PML) (3–7). Moreover, JCPyV could infect the enteric glia and cause chronic idiopathic intestinal pseudo-obstruction (8), or result in male lower urinary tract symptoms (9). The autoimmune diseases of JCPyV-associated brain syndromes include multiple sclerosis (MS), Crohn’s disease, and psoriasis, which were not previously considered as predisposing factors for PML (10). In non-permissive cells (i.e., cells that do not allow viral replication), JCPyV infection causes either abortive infection or malignant transformation (1) (**Figure 2**).

## The infection and replication of JCPyV

**Figure 3:**
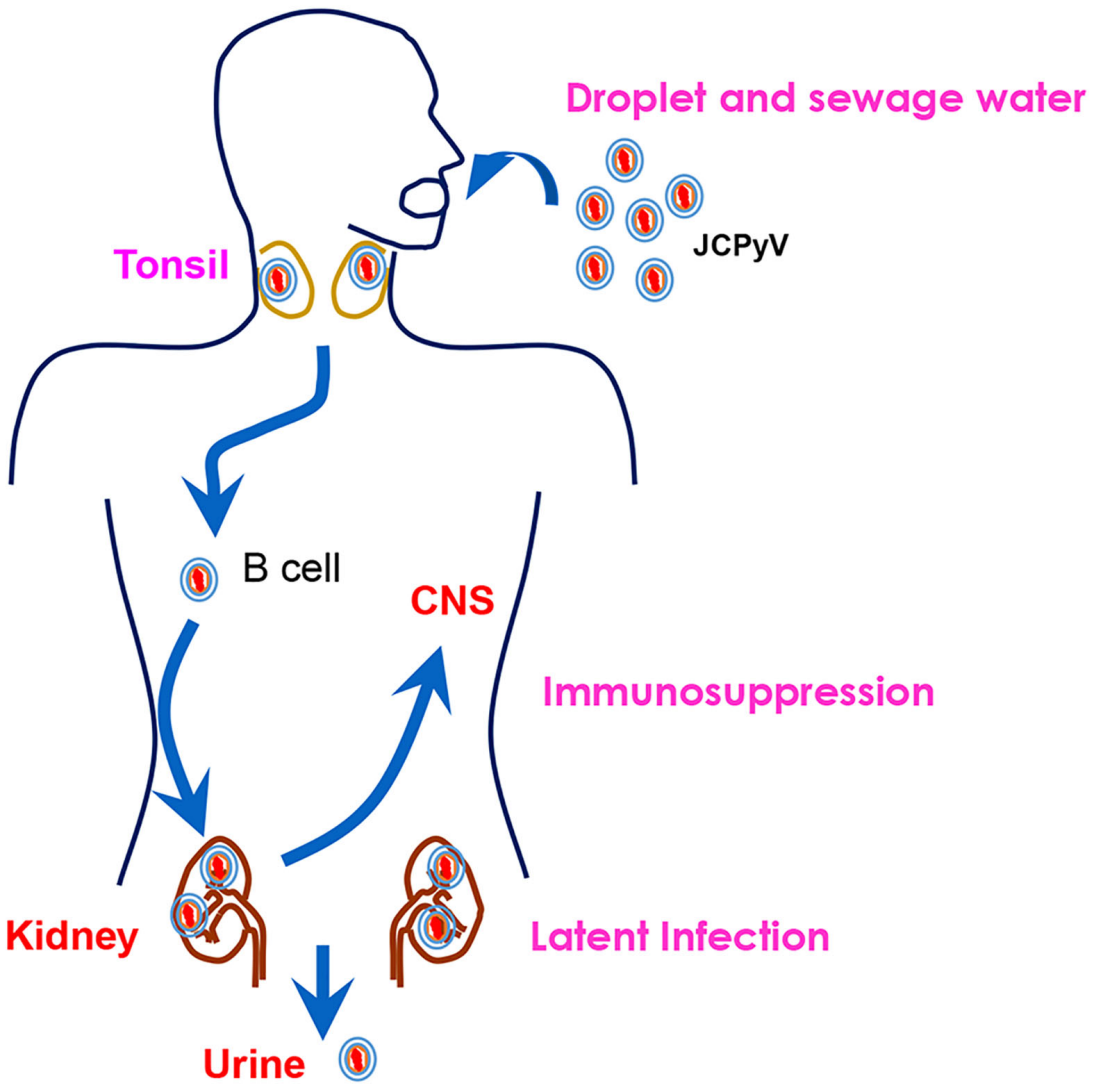
The natural history of JCPyV. JCPyV enters the human body through the intake of raw sewage or inhalation of air droplets. It is transported to the kidneys *via* B cells. It persists quiescently in the tonsil and renal tissues during latency. Upon immunosuppression, JCPyV enters the central nervous system (CNS) and undergoes lytic proliferation, resulting in the demyelinating brain disease, progressive multifocal leukoencephalopathy (PML). It can be excreted from the human body through the urine.

As shown in **Figure 3**, JCPyV enters the human body through the intake of raw sewage or the inhalation of air droplets, and persists quiescently in tonsillar lymphoid and renal tissues during latency (11). Parent-to-child transmission is also common for its propagation (12). After asymptomatic primary infection in childhood, the virus spreads through the bloodstream from the primary sites of infection to secondary sites (kidney and lymphoid tissues, peripheral blood leukocytes, and brain tissue) (13). JCPyV DNA replication occurs primarily in lymphoid and glial cells that contain transcription factors specific for JCPyV (14, 15).

JCPyV infects human cells by the interaction of capsid VP proteins with receptors on JCPyV-sensitive cells, followed by endocytosis and nuclear transport of JCPyV virions. In the nucleus, the viral DNA is uncoated, initiating the transcription of the early region (16). The tissue-specific distribution of the VP receptors (α 2, 6-linked sialic acid, non-sialylated glycosaminoglycans, and serotonin) determines the different infection capabilities of JCPyV (17–21). JCPyV infection is dependent on the interactions between VP capsid proteins and asparagine N-linked sialic acids or the serotonin 5-hydroxytryptamine 2A receptor (5-HT2AR) on the cell surface. Treatment with an inhibitor of N-linked glycosylation (tunicamycin), 5HT2AR antagonists (ketanserin and ritanserin), or anti-5HT2aR antibody reduces JCPyV infection, while treatment with PNGase F to remove N-linked oligosaccharides does not influence JCPyV infection (18, 19, 22). VP1-composed virion-like particles (VLPs) can bind to sialoglycoproteins (α1 acid-glycoprotein, transferrin receptor, and fetuin) and glycolipids (gangliosides and lactosylceramide) (23). Exposure to either anti-VP1 antibody or sialidase to hydrolyze sialic acid residues can suppress viral entry into host cells. After interaction between capsid proteins and their receptors, JCPyV binds to caveolin-1 and undergoes eps15 and Rab5-GTPase-mediated internalization and clathrin-dependent endocytosis (24), which is facilitated by the interaction of β-arrestin with 5-hydroxytryptamine receptors (25).

After the entry of JCPyV into cells, TCF-4-T-antigen complex binds to the JCPyV promoter in U87-MG cells, increasing the ability of the T antigen to replicate viral DNA (26). LCP-1 also interacts with the lytic control element and differentially regulates T antigen expression (27). Glial factor 1 (GF-1) has homology with the central region of Sμbp-2 and can bind to the promoter B-regulatory domain of JCPyV (28). Purα interacts with T antigen to modulate T-antigen-mediated transcriptional activation, while the Purα-BAG-1 complex suppresses JCPyV DNA replication in glial cells (29, 30). The terminal core kinase of the MAPK cascade (MAPK-ERK) facilitates the transcription of the JCPyV by up-regulating the transcription factors downstream of the MAPK cascade (i.e., c-myc and SMAD4) and shuttling them to the nucleus (31), while SF2/ASF (splicing factor 2/alternative splicing factor) weakens the transcription and alternative splicing of JCPyV genes *via* direct interaction with the viral promoter (32), and retinoic acid-inducible gene I (RIG-I) and cGMP-AMP synthase negatively controls JCPyV replication in human astrocytes (33).

Moreover, HIV-1 induces cytokines that reactivate JCPyV to cause PML in the brain, suggesting a direct correlation between inflammatory cytokines and the susceptibility to JCPyV infection (34, 35). The treatment of glial cells with IFNα and IFNβ increases the endogenous levels of C/EBPβ-LIP, which inhibits basal and NF-κB-stimulated JCPyV transcription *via* the NF-κB-C/EBPβ-LIP -JCPyV DNA complex (36). Both TGF-β1 and TNF-α can stimulate JCPyV multiplication and increase the overall number of infected cells *via* the Smad and NF-κB pathways, respectively (37). Tat and Fast1 can cooperate with Smad2, 3, and 4 at the JCPyV DNA control region, stimulating its gene transcription in oligodendroglial cells (38). JCPyV infection significantly increases nuclear HIF-1α levels in glial cells, which binds to and activates the JCPyV early promoter *via* Smad3 and Smad4 (39). IL-1β dramatically increases JCPyV transcription in glial cells *via* NF-1 binding to the JCPyV enhancer region *via* the PKC pathways (40). However, interferon-γ inhibits JCPyV replication by down-regulating T antigen expression *via* Jak1 signaling (41).

According to recent literatures, topoisomerase I inhibitors (β-lapachone and topotecan) are found to inhibit JCPyV infection in neuroblastoma cells (42). Irisolidone, an isoflavone metabolite, negatively modulates JCPyV gene expression by suppressing Sp1 binding in glial cells (24). The Cdk inhibitor, R-roscovitine, suppresses the proliferation and production of JCPyV by inhibiting the phosphorylation of T antigen (43). Hexadecyloxypropyl- cidofovir suppresses JCPyV replication in fetal brain SVG cells (44). JCPyV infection can be suppressed by nocodazole, cytochalasin D, or acrylamide in glial cells (45). Moreover, O’Hara et al. (46) found that teriflunomide could inhibit JCPyV infection and propagation in choroid plexus epithelial cells and glial cells. PARP-1 inhibitor, 3-aminobenzamide, could significantly suppress JCPyV replication and spread (47). In contrast, both trichostatin A (TSA) and butyrate can activate the JCPyV promoter and hyperacetylation of this promoter in non-glial cells. The enhancer and Sp1 element upstream of the TATA box are necessary for TSA-mediated activation (48). Some reagents are expected to prevent the infection and replication of JCPyV in the future.

## The functions of JCPyV-encoded proteins

### T antigen

T antigen is a multifunctional and oncogenic phosphoprotein essential for viral DNA replication in G_2_-arrested cells *via* ATM- and ATR-mediated G2 checkpoint signaling (49). It binds to and breaks DNA to unwind the double helix and recruits helicase, ATPase, and polymerase (1, 50). T antigen primarily targets protein complexes that have PP4 and PP1 phosphatase, v-ATPase, and E3-ubiquitin ligase activities (51). Its N-terminal portion contains LXCXE and J domains, which are necessary for binding and inactivating the Rb family (52) and its N-terminal phosphorylation site at threonine 125 is critical to T-antigen-mediated replication *via* stabilizing T antigen, interaction with the Rb family members p107 and p130 and the release of E2F from RB-E2F complex (53). The origin-binding domain of T antigen contains a C-terminal pocket and interacts with the major groove of GAGGC sequences. The pocket residue increases T antigen expression, supporting JCPyV DNA replication (54).

Reportedly, AP-1 family (c-Fos and Jun) functionally interacts with T antigen, significantly diminishing T-antigen-mediated replication and transcription of JCPyV genes in glial cells. The c-Jun-binding domain for T antigen maps to the middle portion of the protein, while the T-antigen-binding domain for c-Jun is its basic-DNA binding region (55). In glial cells, T antigen interacts with Purα and serine/arginine-rich splicing factor 1 (SRSF1). T antigen promotes JCPyV gene expression by binding to the SRSF1 promoter and weakening SRSF1 transcription (56, 57). Purα and T antigen bind to the JCPyV early promoter *via* T-antigen, ameliorating SRSF1-mediated inhibition of JCPyV gene expression and replication (58). P53 can interact with T antigen, blocking viral DNA replication (59). However, neurofibromatosis type 2 could induce proteasomal degradation of the T-antigen and suppress T-antigen protein expression in glioblastoma cells, weakening T-antigen-mediated regulation of the JCPyV promoter (60), and LIP (liver inhibitory protein) expression also induced the degradation of JCPyV T antigen in transgenic mouse tumor cells (32, 61). The partner proteins modulate the biological functions and protein instability of T antigen, which is involved in carcinogenesis and subsequent progression.

### Agnoprotein

The JCPyV agnoprotein shares 50–60% homology with those of BK and SV40 viruses; however, its carboxyl-terminal region is relatively unique. It is firstly detected on day 3 of JCPyV post-infection, and its levels increase until the late stage of infection, and responsible for virion release and viral propagation (62). Agnoprotein localizes to the endoplasmic reticulum (ER) early in infection and then the plasma membrane late in infection (63). Agnoprotein is 71 amino acids (8kDa) and stably forms dimers and oligomers through its hydrophobic Leu/Ile/Phe-rich (aa 28–39) domain (64). Residues Lys22 to Asp44 may be the transmembrane domain, and the disulfide bond at Cys40 may trigger oligomerization (65). Its basic amino acid residues at positions 8 and 9 determine its viroporin activity (63). In agnoprotein, the major amphipathic α-helix conformation spans amino acids 23–39 of the Leu/Ile/Phe-rich region, while the minor α-helix consists of Leu6 to Lys13 (66). Leu29 and Leu36 of the major amphipathic α-helix are at the dimer interface, keeping the spatial structure and protein stability (67). All three Phe residues are localized to this amphipathic α-helix and mediate protein folding and stability (68). Moreover, agnoprotein primarily targets 501 cellular proteins containing “coiled-coil” motifs. The agnoprotein- host interactions were involved in protein synthesis and degradation, cellular transport, and organelles, including mitochondria, ER-Golgi, and the nucleus. Among the agnoprotein partners, Rab11B, importin, and Crm-1 have been biochemically validated (68).

In nucleus, agnoprotein promotes T antigen binding to the viral origin with indirect interactions with DNA. It contains several potential phosphorylation sites (ser7, ser11, and thr21) that can be phosphorylated by PKC (69). Small t antigen (aa 82-124) also interacts with agnoprotein and PP2A, suppressing the PP2A-mediated dephosphorylation of agnoprotein and promoting JCPyV replication (70). The amino-terminal of agnoprotein can bind to YB-1 and reduce YB-1-mediated gene transcription (71). The interaction of p53 with agnoprotein can lead to p21 expression, causing G_2_/M arrest and sensitizing cells to cisplatin *via* chromosome fragmentation, micronuclei formation, and impaired double-strand DNA break repair activity by up-regulating the expression of the DNA repair proteins (e.g., Ku70 and Ku80) (72).

In cytosol, agnoprotein predominantly localizes to the perinuclear region of JCPyV-infected cells, and colocalizes with the cellular cytoskeletal protein tubulin (73), which is co-precipitated with phosphorylated agnoprotein (74). Suzuki et al. (75) also demonstrated that agnoprotein could directly interact with fasciculation and elongation protein zeta 1 (FEZ1) and microtubules. The interaction dissociated FEZ1 from the microtubules and inhibited FEZ1-facilitated neurite outgrowth. Saxena et al. (76) reported that the mitochondrial targeting sequence and dimerization domain of agnoprotein mediate mitochondrial localization, where agnoprotein decreased the respiration rate, mitochondrial membrane potential, and ATP production while increasing ROS production and Ca^2+^ uptake.

### Caspids

Major coat protein VP1 couples with a minor coat protein (VP2 or VP3). VP2 and VP3 share DNA binding domain, VP1-binding domain, and nuclear localization signal (NLS). The 16 carboxy- terminal and 12 amino-terminal amino acids of VP1 are essential for the assembly of VLPs. Both minor coat proteins and the myristylation site on VP2 are important for properly packaging the genomic DNA of JCPyV (77). Furthermore, the cysteine residues of VP1 are dispensable for protein stability and oligomerization (78, 79). VP1 mediates VLP entry into the nucleus by importins α and β *via* its NLS (80). Point mutations in VP1 can influence virion binding to cellular glycan receptors and their recognition by polyomavirus-specific antibodies (81). Mutation 186G→C (Lys→Asp) in the VP1 gene could predispose MS patients undergoing treatment with natalizumab to PML (82). A deletion of the C-terminal 10 bp of VP1 is closely linked to lytic infection of granule cell neurons and atrophy in the cerebellum of an HIV/PML patient (83). VP1 mutations that are involved binding to sialic acid cell receptors favored PML onset (84). Hsp70 could interact with T antigen and VP2 or VP3, which accumulates in the nucleus of the infected cells and enhances viral DNA replication (85). VP2 binds to DNA through its DNA-binding domain between Lys332 and Lys336 (86). As for capsid expression, Ravichandran et al. (87) found that TGF-β1 activated MEK1/2 and subsequent phosphorylation of Smads, which bound to or increased binding to the JCPyV promoter for VP-1 synthesis.

## The signal pathways of JCPyV

Multi-omics analysis has demonstrated that JCPyV-related carcinogenesis involves aberrant Forkhead box O, AMPK, p53, and PI3K/Akt signaling pathways. Moreover, T antigen can upregulate the expression of Akt, Rb, and survivin and downregulate p21 expression, indicating that it might activate the Akt/NF-κB/survivin pathway to block apoptosis and cause Rb hyper-expression and p21 hypo-expression for cell cycle progression (88). The upregulated proteins are involved in signaling through Cyclin-CDK, TGF-β receptor 1, fibroblast growth factor family receptor and platelet-derived growth factor receptor and the inflammatory responses mediated by Cox-2 (89). T antigen might interact with ribosomal proteins, various keratins, G proteins, apolipoproteins, ubiquitin-related proteins, CCAAT enhancer-binding proteins, β-catenin, RPL19, β-TRCP, and p53 in lens tumor cells (88). T antigen knockdown could suppress glycolysis, mitochondrial respiration, proliferation, migration, and invasion in lens tumor cells; however, it promoted apoptosis. T antigen can also activate the Akt/NF-κB/survivin pathway, producing an anti-apoptosis effect and causing Rb hyperexpression and p21 hypoexpression to mediate cell cycle progression (88). These findings suggest that the T antigen can aggravate the cellular phenotype, possibly by inactivating tumor suppressors, activating oncogenes, or disrupting metabolism and cell adhesion.

**Figure 4:**
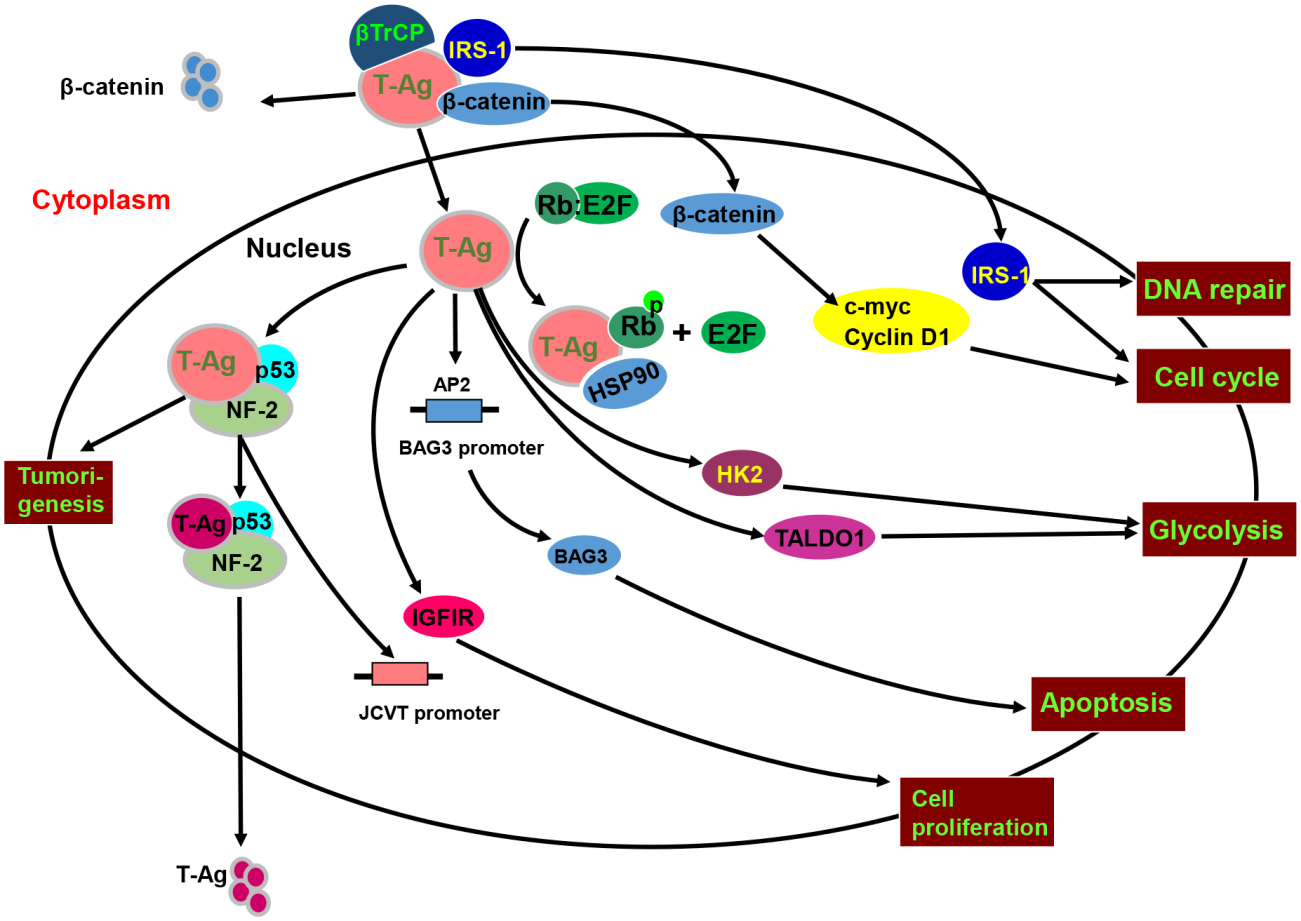
The biological function and signal pathways of the JCPyV T antigen. JCPyV T antigen binds to βTrCP1/2 protein to cause ubiquitin-mediated degradation of β-catenin, and binds to β-catenin to enhance its protein stability and facilitate its entry into the nucleus. Nuclear β-catenin promotes S-phase transformation by up-regulating c-myc and Cyclin D1 protein expression. T antigen interacts with p53 and neurofibromatosis-2 (NF-2) for the proteasome-mediated degradation of T antigen. The binding of T antigen to phosphorylated Rb protein results in the separation of Rb-E2F, resulting in an abnormal cell cycle. T antigen can promote the translocation of the insulin receptor substrate 1(IRS1) to the nucleus, induce cell cycle evolution, and participate in DNA repair. In addition, T antigen up-regulates the expression and phosphorylation of IRS1 and IGF1 receptor (IGF-1R), promoting cell proliferation and disrupting normal cell activity by binding to the transcription factor AP-1. The DNA binding domain of T antigen can bind to the AP2 sequence in the BAG3 promoter and CPG binding protein promoter of transcriptional regulatory methylation and regulate the expression of BAG3. T antigen can also stimulate the expression of transaldolase-1 (TALDO1) and hexokinase-2 (HK2) to induce glycolysis.

As shown in **Figure 4**, p53 interacts with T antigen to repress transcription from the JCPyV early promoter and JCPyV DNA replication in non-glial cells (90, 91). The interaction between p53 and T-antigen up-regulated the p53 downstream target protein, p21/WAF1 (92). Additionally, E2F-1 dissociated from the pRb-E2F-1 complex and stimulated S phase-specific genes following the formation of a pRb-T antigen complex or Rb phosphorylation (93). T antigen can bind to pRb2/p130, p107, and pRb/p105, activating the E2F transcription factor family and promoting entry into S phase (94). As IRS1 signal pathway, T antigen also induces the nuclear translocation of IRS-1, and IRS-1 interacts with T antigen, which is independent of IRS-1 tyrosine phosphorylation and blocked by IRS-1 serine phosphorylation (95). After T-antigen-mediated nuclear translocation, IRS-1 binds to Rad51 at the site of damaged DNA to direct DNA repair, causing accumulation of mutations in the affected cells (96). IRS-1-Rad51 nuclear interaction also sensitizes JCV T-antigen positive medulloblastoma cells to cisplatin and γ-irradiation (97). T-antigen requires the presence of a functional insulin-like growth factor I receptor (IGF-IR) for transformation of fibroblasts and for survival of medulloblastoma cell line. IGF-IR is phosphorylated in medulloblastoma biopsies and JCV T-antigen inhibits homologous recombination-directed DNA repair, causing accumulation of mutations. In Wnt- β-catenin pathway, the interaction between the central domain (residues 82–628) of T-antigen and the C-terminal residues of β-catenin (aa 695-781) increases β-catenin levels and its nuclear entry, resulting in the upregulation of its downstream genes (c-myc, VEGF, and Cyclin D1). T antigen binds to the F-box proteins β-transducin repeat-containing protein-1 and 2 (βTrCP1/2) and recruits Rac1 to form the T antigen-Rac1-β-catenin complex that suppresses the ubiquitin- dependent degradation of β-catenin by proteasomes (98–100). T antigen downregulates BAG-3 expression to inhibit apoptosis by blocking AP2 binding to the BAG3 promoter. Bag3 interacts with the T antigen, inducing its autophagic degradation (101). Additionally, T antigen binds to and activates the survivin promoter, upregulating its expression and mediating the nuclear translocation of suvivin *via* the T-antigen-survivin complex (102, 103). In contrast, T antigen can arrest G_1_, sustain G_2_, and block ROS induction and cytotoxicity during glucose deprivation. T antigen can also stimulate the expression of transaldolase-1 and hexokinase-2 (104).

## The association between JCPyV and carcinogenesis

In transformed cells, JCPyV can cause anchorage-dependent growth, rapid division, prolonged life span, increased ploidy, unstable multicentric chromosomes, centric and acentric rings, dysregulated genomic stability and DNA repair, and increased micronuclei formation (105–107). Intracranially inoculated JCPyV caused glioblastoma in juvenile owl monkeys (108), grade 3-4 astrocytoma in adult owl monkeys (109), undifferentiated neuroectodermal tumors in the cerebrums of newborn Sprague-Dawley rats (110), cerebellar medulloblastoma, plexus tumors, medulloblastoma, and thalamic gliomatosis in hamsters (111), and neuroblastoma in the abdominal cavity, pelvis, mediastinum, and neck region of Syrian hamsters (112). Padgett et al. (113) demonstrated that malignant brain tumors developed in Syrian golden hamsters during a 6.5-month observation period following intracerebral inoculation of different JCPyV strains into newborns. The Mad-2 strain caused cerebellar medulloblastomas, whereas the MAD-3 strain induced extracranial neuroblastomas. In contrast, the Mad-4 strain produced pineal gland and cerebellar tumors.

The spontaneous tumors in the transgenic mice of JCV T antigen can provide direct evidences for the oncogenic role of JCPyV as shown in **Table 1**. The transgenic mouse with the early encoding region of the archetype strain was generated using its own promoter and developed neural crest tumors, such as primitive neuroectodermal tumors, adrenal neuroblastomas, medulloblastomas, pituitary tumors, glioblastomas, and malignant peripheral nerve sheath tumors (114). Krynska et al. (115) established the same transgenic mice and observed primitive tumors originating from the cerebellum and adjacent brain stem that were grossly and histologically similar to human medulloblastoma and primitive neuroectodermal tumors. However, Gordon et al. (92) used the same promoter to generate transgenic mice overexpressing T antigen, which developed large, solid pituitary masses. Shollar et al. (116) established transgenic mice expressing T-antigen under the control of the Mad-4 promoter and observed pituitary tumors by one year of age. Krynska et al. (93) found that transgenic mice harboring T antigen could develop massive abdominal tumors of neural crest origin. In our group, a transgene with the K19 promoter was generated and pulmonary tumors with T-antigen, p53, and CK19 expression and EGFR mutation were observed (117). We also established T antigen-expressing transgenic mice using α-crystallin A and observed lens tumors that were positive for T antigen, N-cadherin, p53, and β-catenin. Enlarged eyeballs were observed, and the tumors invaded the brain (118). Additionally, we generated CAG-loxp-LaZ-loxp T antigen transgenic mice with T antigen activation induced using matching tissue-specific cre transgenic mice. Gastric poorly-differentiated carcinoma was observed in gastric stem-like and chief cells following T antigen overexpression. Moreover, spontaneous hepatocellular and colorectal cancers developed in Alb-cre (hepatocytes)/T antigen and villin-cre (intestinal epithelium)/T antigen transgenic mice. Gastric, colorectal, and breast cancer were observed in PGC (Pepsinogen C)-cre/T antigen mice. Pancreatic insulinoma and ductal adenocarcinoma, gastric adenoma, and duodenal cancer were detected in Pdx1-cre/T antigen mice. There was alternative splicing of T antigen mRNA in all target organs of these transgenic mice and various cells transfected with pEGFP-N1-T antigen. It has been suggested that the JCPyV T antigen might induce gastroenterological carcinogenesis in a cell-specific manner (119).

**Table 1:** The JCPyV T antigen-induced spontaneous tumor in the transgenic mice.

Author and reference	Promoter name	Tissue and cellular specificity of promoter	Cancer types
Gordon et al. (92)	viral own promoter of Mad1	no	Pituitary tumors
Krynska (93)	viral own promoter of Mad1	no	Abdominal tumors of neural crest origin d
Del Valle et al. (114)	viral own promoter of Mad1	no	primitive neuroectodermal tumors, medulloblastomas, adrenal neuroblastomas, pituitary tumors, malignant peripheral nerve sheath tumors, and glioblastomas
Krynska et al. (115)	viral early region of Mad1	no	primitive invasive tumors originating from the cerebellum and the surrounding brain stem
Shollar et al. (116)	viral control region of the Mad-4 promoter	no	pituitary tumors, solid masses around the salivary gland, the sciatic nerve, and peripheral nerve sheath tumors
Noguchi et al. (117)	cytokeratin 19 promoter	gastric stem-like cells	lung adenoma and adenocarcinoma
Gou et al. (118)	α-crystallin A promoter	lens epithelium	lens tumors
Zheng et al. (119)	Albumin promoter	hepatocyte	Hepatocellular carcinoma
	villin promoter	intestinal epithelium	colorectal cancer
	cytokeratin 19 promoter	gastric stem-like cells	gastric cancer
	PGC promoter	gastric chief cells	gastric cancer, breast cancer
	Pdx1 promoter	pancreas and duodenum	pancreatic adenocarcinoma, insulinoma, vater’s cancer, gastric tumors

PGC, pepsinogen C; Pdx1, pancreas/duodenum homeobox protein 1.

It is important to detect and compare JCPyV DNA in cancer and adjacent normal tissues using either molecular or morphological approach. JCPyV detection might determine the etiology for JCPyV-related cancer. The correlation of JCPyV T antigen with carcinogenesis and subsequent progression was summarized in **Table 2**. Although JCPyV DNA was found in ependymomas and choroid plexus papilloma (138), Kutsuna et al. (120) found that glossitis and tongue dysplasia had significantly lower copies of JCPyV than tongue cancer. They observed T antigen DNA and protein in the nuclei of tongue cancer cells but not in normal or dysplastic epithelia. JCPyV DNA and T antigen were found in adenoid cystic carcinomas samples of the trachea, paranasal sinuses, and oral cavity by PCR and immunohistochemistry respectively (121). JCPyV DNA was more frequently detected in esophageal carcinomas than in normal, benign, or premalignant esophageal samples (122). JCPyV T antigen load is also higher in gastric cancer than in normal mucosa (123). Indeed, its DNA and protein were detected in the nuclei of gastric cancer cells. Moreover, T-antigen DNA is correlated with differentiation and the methylation of p14 and p16 in this cancer (124).

**Table 2:** The correlation of JCPyV T antigen with carcinogenesis and subsequent progression.

Author and references	Cancer type	DNA profile	Protein profile	Clinical and prognostic significances
Kutsuna et al. (120)	Oral squamous carcinoma	high	nd	ns
Hämetoja et al. (121)	adenoid cystic carcinoma of the oral cavity and the airways	nd	nd	ns
Del Valle et al. (122)	Esophageal squamous carcinoma	high	nd	ns
Murai et al. (123)	Gastric cancer	high	nd	ns
Ksiaa et al. (124)	Gastric cancer	high	nd	positively associated with elder age, differentiation, hypermethylation of p14 and p16 and poor prognosis.
Hori et al. (125)	Colorectal cancer	high	high	ns
Shavaleh e t al (126).^*^	Colorectal cancer	high	ns	ns
Nosho et al. (127)	Colorectal cancer	high	nd	negatively associated with proximal location, high grade, family history of colorectal cancer, and mucinous component and was associated with p53 expression, high CIN score, Cyclin D1 expression, LINE-1 hypomethylation, and BRAF mutation
Link et al. (128)	Colorectal cancer	high	nd	positively associated with clinical staging and liver metastasis
Vilkin et al. (129)	Colorectal cancer	nd	nd	positively associated with hMLH1 hypermethylation
Ripple et al. (130)	Colon cancer	nd	high	negatively associated with β-catenin expression**
Abdel-Aziz HO (131)	Lung cancer	high	nd	positively associated with lymph node metastasis, p53 and nuclear β-catenin expression, and high in adenocarcinoma than squamous carcinoma
Zheng et al. (132)	Lung cancer	high	high	positively associated with ki-67 and no membrane β-catenin expression, and high in adenocarcinoma than squamous carcinoma, small and large cell carcinoma
Antje et al. (133)	Renal clear cell carcinoma	nd	nd	ns
Shen et al. (134)	Prostate cancer	high	high	positively associated with PSA level and Gleason’s scores
Zheng et al. (135)	Breast cancer	low	low	negatively correlated with tumor size, E-cadherin expression and triple-negative breast cancer, but positively correlated with lymph node metastasis, histological grading and ER and PR expression.
Zheng et al. (136)	Hepatocellular carcinoma	low	high	ns
Zheng et al. (136)	Pancreatic cancer	low	high	ns
Zheng et al. (137)	Cervical cancer	high	nd	ns

nd, not detection; ns, not significant; ER, estrogen receptor; PR, progestogen receptor; PSA, prostate-specific antigen; *meta-analysis; **protein level.

In colorectal cancer, the positivity rate of T antigen is decreased from colorectal adenocarcinoma to adenoma to mucosa (125, 126). Nosho et al. (127) reported that T antigen could inactivate wild-type p53, resulting in chromosomal instability. It was positively correlated with p53 expression, p21 loss, nuclear β-catenin, LINE-1 hypo-methylation and hyper-expression, and low MSI (microsatellite instability) levels. Link et al. (128) found that T antigen enhanced the migration and invasion of colorectal cancer cells *via* Akt and MAPK signaling. Indeed, T antigen could be detected by IHC in primary colorectal cancers and their corresponding liver metastases. The interaction between T-antigen and β-catenin and the nuclear detection of β-catenin in T-antigen-positive colorectal cancer cells demonstrates dys-regulation of the Wnt pathway (15, 129). Ripple et al. (130) found that T antigen and β-catenin were co-localized in the nuclei of colorectal cancer cells, resulting in the activation of TCF4-dependent promoters and the transcription of TCF4 downstream targets (e.g. c-myc, VEGF and Cyclin D1).

In the respiratory cancer, the positivity rate for the JCPyV T antigen in the respiratory system is lower in normal lung tissue than in tumors; T antigen DNA is strongly observed in lung adenocarcinoma (131). One study found a lower JCPyV copy number in normal lung cancer than in lung tumors (132). Moreover, the copy number was lower in lung adenocarcinomas compared to squamous, small, or large cell carcinomas. Lung cancers with a high JCPyV copy number were characterized by high proliferation and low β-catenin-mediated cell adhesion (132).

In urinary tract neoplasms, JCPyV has also been detected in renal pelvic urothelial carcinoma and renal cell carcinoma (133). Shen et al. (134) found that 90.1% of the urothelial carcinomas and all the renal cell carcinomas that they evaluated were positive for JCPyV using nested PCR. Prostate cancer is more susceptible to JCPyV infection than benign prostate hyperplasia. Tumors with both high prostate-specific antigen levels and high Gleason scores were associated with a high risk of JCPyV infection.

In addition, we reported that the positivity rate and expression levels of T antigen were lower in breast cancer than in normal breast tissue (135), in line with hepatocellular and pancreatic cancer (136). T antigen DNA positivity was inversely associated with E-cadherin expression and triple-negative breast cancer but positively associated with lymph node involvement and ER and PR expression. JCPyV copies were negatively linked to tumor size and E-cadherin expression in breast cancer but positively associated with histological grading. Additionally, we for the first time found that JCPyV was less detectable in cervical epithelium than dysplasia and carcinoma (137). We also observed T antigen DNA and protein in hepatocellular, pancreatic, breast and cervical cancer cells using *in situ* PCR and immunohistochemistry (134–136).

## Conclusions and perspective

JCPyV enters eukaryotic cells and is inserted into genomic DNA. It induces tumorigenesis with tissue specificity by targeting the p53, β-catenin, IRS, Rb, TGF-β1, PI3K/Akt, and AMPK signal pathways. Pathological examination and animal experiments have demonstrated that the JCPyV T antigen might induce tumorigenesis in neural and gastroenterological systems and breast. Thus, JCPyV might be an etiological risk factor for carcinogenesis and should be emphasized in tertiary prevention and treatment of cancer.

Because JCPyV infection rate reaches 80%, we should try our best to prevent the entry of JCPyV into the human body through the sewage and air droplet. In addition, it is better to block the endocytosis and nuclear transport of JCPyV virions by receptor antagonists. Finally, the agents to block the JCPyV infection or inhibit the JCPyV-related signal pathway should be developed to prevent and treat JCPyV-related cancers. In the future, we can realize the early diagnosis, finding and treatment of JCPyV-related cancers.

## Author contributions

H-CZ conceived the review, and HX and CY-Z led its drafting and managed the editing of it. All authors contributed to the drafting of the review and approved the final manuscript.

## Funding

This review was supported by Award for Liaoning Distinguished Professor, Natural Science Foundation of Hebei Province (21377772D) and National Natural Scientific Foundation of China (81672700).

## Conflict of interest

The authors declare that the research was conducted in the absence of any commercial or financial relationships that could be construed as a potential conflict of interest.

## Publisher’s note

All claims expressed in this article are solely those of the authors and do not necessarily represent those of their affiliated organizations, or those of the publisher, the editors and the reviewers. Any product that may be evaluated in this article, or claim that may be made by its manufacturer, is not guaranteed or endorsed by the publisher.
